# The *N*‐acetylglucosamine catabolic gene cluster in *Trichoderma reesei* is controlled by the Ndt80‐like transcription factor RON1

**DOI:** 10.1111/mmi.13256

**Published:** 2015-11-19

**Authors:** Lisa Kappel, Romana Gaderer, Michel Flipphi, Verena Seidl‐Seiboth

**Affiliations:** ^1^ Research Division Biotechnology and MicrobiologyInstitute of Chemical EngineeringTU WienViennaAustria; ^2^ Department of Biochemical Engineering, Faculty of Science and TechnologyUniversity of DebrecenDebrecenHungary

## Abstract

Chitin is an important structural constituent of fungal cell walls composed of *N*‐acetylglucosamine (GlcNAc) monosaccharides, but catabolism of GlcNAc has not been studied in filamentous fungi so far. In the yeast *C* *andida albicans*, the genes encoding the three enzymes responsible for stepwise conversion of GlcNAc to fructose‐6‐phosphate are clustered. In this work, we analysed GlcNAc catabolism in ascomycete filamentous fungi and found that the respective genes are also clustered in these fungi. In contrast to *C* *. albicans*, the cluster often contains a gene for an Ndt80‐like transcription factor, which we named RON1 (regulator of *N*‐acetylglucosamine catabolism 1). Further, a gene for a glycoside hydrolase 3 protein related to bacterial *N*‐acetylglucosaminidases can be found in the GlcNAc gene cluster in filamentous fungi. Functional analysis in *T* *richoderma reesei* showed that the transcription factor RON1 is a key activator of the GlcNAc gene cluster and essential for GlcNAc catabolism. Furthermore, we present an evolutionary analysis of Ndt80‐like proteins in Ascomycota. All GlcNAc cluster genes, as well as the GlcNAc transporter gene *ngt1*, and an additional transcriptional regulator gene, *csp2*, encoding the homolog of *N* *eurospora crassa* CSP2/GRHL, were functionally characterised by gene expression analysis and phenotypic characterisation of knockout strains in *T* *. reesei*.

## Introduction

Chitin is a linear polysaccharide composed of β‐(1,4) linked *N‐*acetylglucosamine units (GlcNAc; 2‐acetamido‐2‐deoxy‐D‐glucopyranose). In filamentous fungi, chitin is located in the inner layers of the cell wall, close to the plasma membrane (Ruiz‐Herrera, 1991) and forms, together with β‐(1,3–1,6) glucan, the structural scaffold of the fungal cell wall (Latgé, 2007). In the biosphere, chitin is not only found in fungal cell walls, but also in the exoskeletons of protists and arthropods, e.g. insects and shrimps. Chitin is the second most abundant biopolymer after cellulose with a natural turnover of at least 10^9^ tons per year (Muzzarelli, 1999). Nonetheless, chitin does not visibly accumulate in the biosphere, which is indicative for its efficient natural recycling by microbes. Analysis of fungal genomes revealed that filamentous fungi feature a large repertoire of extracellular chitinolytic enzymes. They have typically between 10 and 35 different chitinases belonging to glycoside hydrolase (GH) family 18 and two extracellular GH 20 β‐*N*‐acetylglucosaminidases (chitobiases) that contribute to the degradation of chitin to GlcNAc monomers (Seidl, 2008). The genomes of fungi that parasitise other fungi (mycoparasites) or insects (entomopathogens), e.g. *Trichoderma* species and several entomopathogens, including *Metarhizium anisopliae*, *Beauveria bassiana* and *Cordyceps militaris*, are particularly enriched in chitinase genes (Seidl *et al*., 2005; Gao *et al*., 2011; Kubicek *et al*., 2011; Zheng *et al*., 2011; Xiao *et al*., 2012; Agrawal *et al*., 2015). Chitinases are not only involved in the breakdown of extracellular chitin, but also in self‐digestive processes such as cell wall remodeling during fungal growth and asexual development, as well as in cell wall degradation during autolysis and apoptosis. The roles of chitinases in these processes, and their gene expression, have been studied in several fungal species, e.g. *Aspergillus nidulans* (Pusztahelyi *et al*., 2006; Emri *et al*., 2008; Pócsi *et al*., 2009; Shin *et al*., 2009), *Neurospora crassa* (Tzelepis *et al*., 2012), *Trichoderma atroviride* and *T. virens* (Gruber and Seidl‐Seiboth, 2012), and *Penicillium chrysogenum* (Sámi *et al*., 2001; Kamerewerd *et al*., 2011; Pusztahelyi and Pócsi, 2014). The data suggest that several chitinases have dual roles and are involved in the degradation of extracellular chitin as well as remodeling and recycling of cell wall chitin during different growth stages (Gruber and Seidl‐Seiboth, 2012). From this broad spectrum of different chitinases, the pathway of chitin degradation narrows down to one or two *N*‐acetylglucosaminidases (Seidl, 2008). In *T. atroviride*, it was shown that the presence of either one of these two *N*‐acetylglucosaminidases is essential for extracellular conversion of chitobiose to GlcNAc and for growth on chitin (López‐Mondéjar *et al*., 2009). Uptake and intracellular catabolism of GlcNAc have scarcely been studied in fungi to date, with the notable exception of the human pathogenic diploid yeast *Candida albicans* (Kumar *et al*., 2000; Alvarez and Konopka, 2007; Biswas *et al*., 2007; Naseem *et al*., 2011; 2015; Konopka, 2012; Rao *et al*., 2013). *Saccharomyces cerevisiae* and *Schizosaccharomyces pombe* lack the genes needed to catabolise GlcNAc.

**Figure 1:**
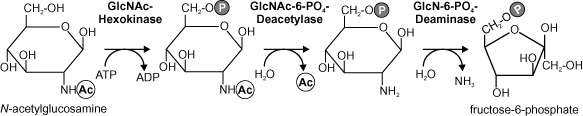
Schematic representation of the GlcNAc catabolism pathway. GlcNAc is phosphorylated by GlcNAc hexokinase, then GlcNAc‐6‐phosphate is deacetylated by GlcNAc‐6‐phosphate deacetylase, and subsequently, GlcN‐6‐phosphate deaminase converts GlcN‐6‐phosphate into fructose‐6‐phosphate. Acetate and ammonium are the other end products of GlcNAc catabolism.

In *C. albicans*, GlcNAc is a potent inducer of hyphal growth related to the virulence of this dimorphic human pathogen (Naseem *et al*., 2011; Ishijima *et al*., 2012; Rao *et al*., 2013). GlcNAc stimulates the switch from budding to hyphal growth and induces the expression of virulence genes, but it also induces the expression of the genes needed to catabolise GlcNAc. To induce signaling, GlcNAc must enter *C. albicans* cells, although stimulation of hyphal growth does not require GlcNAc catabolism (Naseem *et al*., 2011; 2015). Transport of GlcNAc in *C. albicans* is mediated by the plasma membrane transporter Ngt1, which belongs to the large Major Facilitator Superfamily (Alvarez and Konopka, 2007). The three genes encoding the enzymes necessary for GlcNAc catabolism are organised in a gene cluster that consists of adjacent genes encoding a sugar kinase (Nag5/Hxk1) (EC 2.7.1.59) that phosphorylates GlcNAc to create GlcNAc‐6‐phosphate, a deacetylase (Nag2/Dac1) (EC 3.5.1.33) that splits it into acetate and glucosamine‐6‐phosphate, and a deaminase (Nag1) (EC 3.5.99.6) that subsequently converts glucosamine‐6‐phosphate to ammonium and fructose‐6‐phosphate, an intermediate of glycolysis (Kumar *et al*., 2000; Naseem *et al*., 2011) (Fig. 1). Deletion mutants of *HXK1*, *NAG1* and *DAC1* failed to utilise GlcNAc as carbon source, and surprisingly, GlcNAc inhibited the growth of *NAG1* and *DAC1* deletion mutants on other sugars (D‐glucose, D‐fructose, D‐galactose), suggesting that excess GlcNAc‐6‐phosphate is deleterious (Naseem *et al*., 2011). However, the transcriptional regulator of the *C. albicans* GlcNAc catabolic pathway has not been identified so far.

In this study, we analysed the genomic organisation of the GlcNAc catabolic gene cluster in ascomycete filamentous fungi. We found that it often contains a gene for a transcription factor with an Ndt80‐like DNA‐binding domain. Expression of this transcription factor and the four structural GlcNAc catabolism genes was analysed in two species of *Trichoderma*, and the genes were functionally characterised upon generation of knockout strains in *T. reesei*.

## Results

### Organisation of the GlcNAc catabolic gene cluster in filamentous fungi

**Figure 2:**
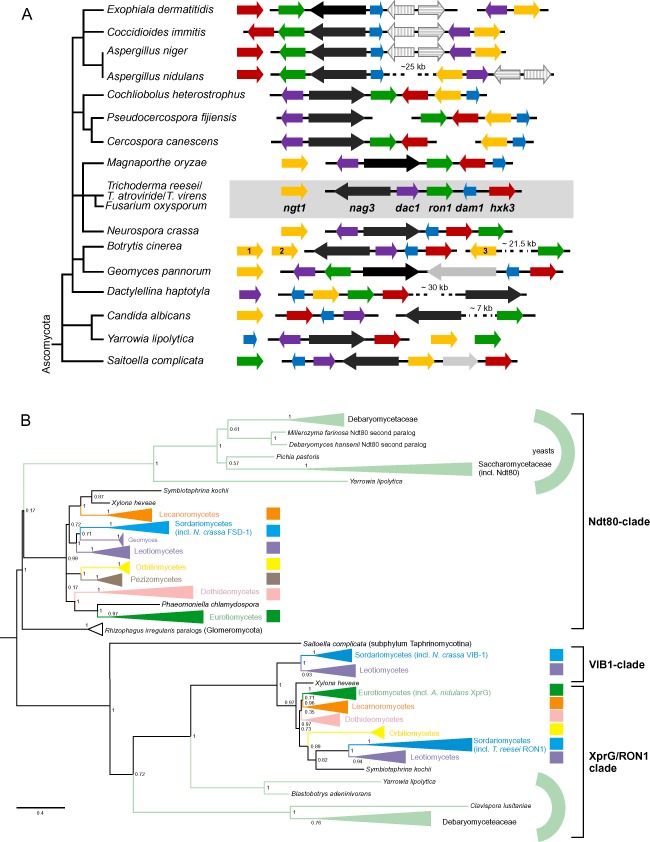
(A) Genomic organisation of GlcNAc gene clusters in Ascomycota. GlcNAc cluster genes are depicted as coloured arrows to indicate their identity and orientation: *nag3*, black; *ngt1*, yellow; *hxk3*, red; *dac1*, purple; *dam1*, blue; *ron1*, green. Gene clustering is indicated by arrows superposed on an uninterrupted, thin horizontal black bar. Intergenic spaces are not drawn to scale. Unlinked genes involved in GlcNAc catabolism are always depicted with the arrowhead (3′ end) pointing to the right. Single genes unlikely to be involved in GlcNAc catabolism but nevertheless located between GlcNAc cluster genes in certain species are represented by grey arrows. Evolutionary relations among the 18 species are reflected by the schematic, flattened ‘taxonomical tree’ at the left. (B) Phylogenetic analysis of putative Ndt80 family proteins in Ascomycota. Data were condensed at the level of classes (Color code: Orbiliomycetes, yellow; Pezizomycetes, brown; Lecanoromycetes, orange; Leotiomycetes, purple; Sordariomycetes, blue; Eurotiomycetes, dark green; and Dothideomycetes, pink), while for Saccharomycotina, branches were collapsed at the level of families (essentially, Saccharomycetaceae – type species: *S* *. cerevisiae* – and Debaryomycetaceae – type species: *C* *. albicans*). To facilitate recognition, branches of yeast proteins are depicted in light green shades.

Fungal genomes were screened for the presence of homologs of the GlcNAc catabolic cluster genes and the GlcNAc transporter from *C. albicans*. The results showed that the respective genes are also clustered in ascomycete filamentous fungi and that a homolog of the *C. albicans* transporter gene *ngt1* can also be found in these genomes (Fig. 2A). Considering extant gene names in *Trichoderma* and other filamentous fungi (e.g. hexokinases *hxk1* and *hxk2*, β‐*N*‐acetylglucosaminidases *nag1* and *nag2*), the structural genes of the GlcNAc catabolic cluster were designated *hxk3* (GlcNAc‐hexokinase), *dac1* (GlcNAc‐6‐phosphate deacetylase), *dam1* (glucosamine‐6‐phosphate deaminase) and *ngt1* (GlcNAc:H^+^ symporter) respectively.

In filamentous fungi, besides these genes encoding the enzymes necessary for the stepwise conversion of GlcNAc into fructose‐6‐phosphate (see Fig. 1) and its transporter, a gene encoding a GH family 3 protein can often be found in the cluster, which we have annotated as *nag3*. NAG3 exhibits similarity to bacterial GH3 β‐*N*‐acetylhexosaminidases (Tsujibo *et al*., 1994; Cheng *et al*., 2000; Li *et al*., 2002; Mayer *et al*., 2006; Litzinger *et al*., 2010) and http://www.cazy.org/GH3.html). In fungi, *N*‐acetylglucosaminidases have so far only been described from GH family 20 (Seidl, 2008; López‐Mondéjar *et al*., 2009). Recently, it was reported for Nag3 from *Cellulomonas fimi* that the GH3 enzyme is actually a GlcNAc‐phosphorylase using phosphate rather than water as nucleophile (MacDonald *et al*., 2015).

Interestingly, our analysis showed that the GlcNAc cluster in filamentous fungi in many cases also contains a gene for a transcription factor with an Ndt80‐like DNA‐binding domain (PFAM family PF05224). The transcription factor was designated RON1 (**r**egulator **o**f ***N*** *‐*acetylglucosamine catabolism 1). RON1 belongs to a rare family of exclusively fungal transcription factors, epitomised by *S. cerevisiae* Ndt80 (Xu *et al*., 1995; Chu and Herskowitz, 1998; Fingerman *et al*., 2004; Lamoureux and Glover, 2006). The genome of the filamentous model fungus *N. crassa* contains three genes encoding proteins with an Ndt80‐like DNA‐binding domain (Hutchison and Glass, 2010). While in *S. cerevisiae* Ndt80 is primarily involved in the regulation of meiosis, the Ndt80‐like proteins in *N. crassa* (VIB‐1, FSD‐1) have other functions. Mutations in *fsd‐1* affected the timing and development of female reproductive structures and ascospore maturation (Hutchison and Glass, 2010), and the *A. nidulans* homolog, *ndtA*, is apparently required for sexual reproduction (Katz *et al*., 2013). Further, VIB‐1 is involved in the regulation of vegetative incompatibility and programmed cell death. In addition, VIB‐1 is a major regulator of responses to nitrogen and carbon starvation, involved in protoperithecial development, and was recently reported to be essential for plant cell wall degradation by repressing glucose signalling and carbon catabolite repression (Dementhon *et al*., 2006; Hutchison and Glass, 2010; Xiong *et al*., 2014). For the third transcription factor in *N. crassa* belonging to the Ndt80‐family (encoded by the gene at locus NCU04729), no phenotype or overlap in function with either *fsd‐1* or *vib‐1* has been reported (Hutchison and Glass, 2010). We have now found that the gene at locus NCU04729 is part of the GlcNAc catabolic cluster of *N. crassa* (Fig. 2A). In *A. nidulans*, classical loss‐of‐function mutations in the Ndt80 transcription factor gene *xprG*/*phoG* (initially called *pacG*) resulted in the loss of an acid phosphatase (Caddick and Arst, 1986) and in decreased extracellular protease production in response to carbon and nitrogen starvation (Katz *et al*., 2006). In *A. nidul*ans, the *xprG* gene is closely linked to three of the structural GlcNAc catabolic genes and neighbours the *nag3* ortholog (Fig. 2A), but the cluster is effectively split in two as a consequence of a chromosomal inversion.

In order to elucidate the evolutionary relationships among Ndt80‐like transcription factors in filamentous fungi (Pezizomycotina), a phylogenetic analysis was performed, including some 500 proteins from over 200 species of Ascomycota. Figure 2B shows a condensed phylogenetic tree summarising our findings. A more detailed species‐annotated tree of the same analysis can be found in Fig. S1. The results indicated that Ndt80‐like proteins can be assigned to two groups that likely existed in a common ancestor of all Ascomycota. One superbranch contains direct orthologs of *S. cerevisiae* Ndt80, and includes members from many species belonging to the two subphyla Pezizomycotina and Saccharomycotina. A characterised filamentous fungal member of this superbranch is *N. crassa* FSD‐1. *N. crassa* VIB‐1 and *A. nidulans* PhoG/XprG‐like proteins cluster in the other superbranch that constitutes two separate clades. One clade (termed VIB‐1 clade in Fig. 2B) appeared exclusively in the classes Sordariomycetes (including *Trichoderma* spp.) and Leotiomycetes, while the other clade (XprG/RON1 clade in Fig. 2B) featured orthologs from all but one class of Pezizomycotina. Thus, this analysis shows that RON1 and XprG are orthologs that can be found throughout many different classes of Ascomycota, while VIB‐1‐like proteins are RON1‐paralogs unique to Sordariomycetes and Leotiomycetes.

In general, these two clades (VIB‐1‐clade and RON1/XprG‐clade) appear to have evolved in Pezizomycotina after the divergence from Saccharomycotina, including the early divergent yeasts *Yarrowia lipolytica* and *Blastobotrys adeninivorans*, both of which feature a complete set of GlcNAc‐cluster genes (data not shown). Five out of the six GlcNAc cluster genes (including the designate Ndt80‐family regulator) are neither present in the 14 species of Saccharomycetaceae nor in *Pichia pastoris*, which suggests that the GlcNAc cluster has been lost independently from this yeast lineage.

### Expression of the GlcNAc cluster genes is upregulated during growth on GlcNAc and chitin

**Figure 3:**
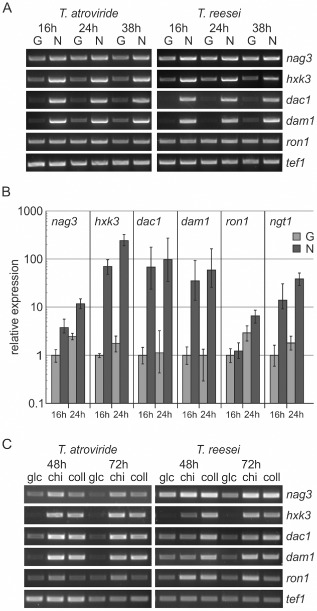
Transcript levels of GlcNAc catabolic cluster genes are strongly increased during growth on GlcNAc. A. Gene expression analysis (semi‐quantitative RT‐PCR) of GlcNAc cluster genes in *T* *. atroviride* and *T* *. reesei* after growth on 1% glucose (G) or 1% GlcNAc (N) for 16 h, 24 h and 38 h. As reference gene *tef1* was used. B. Quantitative gene expression analysis (qRT‐PCR) of the GlcNAc cluster genes in *T* *. reesei*. Samples were normalised to *tef1* expression levels and compared with the time point ‘glucose, 16 h’, which was set as 1 for the respective genes that were studied. The standard deviation of the mean expression values from at least two independent biological replicates is shown; *P* values are < 0.001 except for *hxk3* and *ngt1* (*P* < 0.01 for glucose, 24 h). *ron1* levels at 16 h induction with GlcNAc and *dac1* and *dam1* levels at 24 h on glucose are statistically not significantly different from glucose levels at 16 h. C. Expression of GlcNAc cluster genes in *T* *. atroviride* and *T* *. reesei* after 48 h and 72 h of growth on the complex carbon sources chitin (1%, chi) and colloidal chitin (1%, coll) in comparison to growth on glucose (1%, glc). As reference gene, again, *tef1* was used.

Gene regulation of chitinolytic enzymes and the use of chitin and *N‐*acetylglucosamine as carbon sources have already received considerable attention in *Trichoderma* spp. In order to complement the currently available knowledge with data about GlcNAc catabolism, we decided to analyse the gene expression of the six GlcNAc cluster genes in *T. reesei* and *T. atroviride. T. atroviride* is a mycoparasite and has been the subject of several detailed studies on chitinase and *N*‐acetylglucosaminidase gene regulation. *T. reesei* is a saprotroph and is widely used for biotechnological applications due to its remarkable cellulolytic potential. It is more amenable to molecular genetic manipulations than *T. atroviride*, and a range of different selection markers for gene deletion is readily available for *T. reesei* (Guangtao *et al*., 2009; Seiboth *et al*., 2011; Bischof and Seiboth, 2014). It has been established that GlcNAc is an efficiently consumable carbon source that promotes strong and fast growth of either species (Druzhinina *et al*., 2006; Seidl *et al*., 2006). Chitin, even when used in a pretreated, acid‐hydrolysed form, allows only slow growth and weak biomass formation due to its recalcitrant polymer structure (López‐Mondéjar *et al*., 2009; Gruber *et al*., 2011). For gene expression analysis of the GlcNAc cluster genes, biomass samples were taken from shake flask cultivations of *T. atroviride* and *T. reesei* in minimal medium containing 1% GlcNAc or glucose (control) as the carbon source at 16 h, 24 h and 38 h after inoculation. In both *T. reesei* and *T. atroviride*, *hxk3*, *dac1* and *dam1*, which encode the enzymes of GlcNAc catabolism, exhibited different basal expression levels on glucose, as well as a clear up‐regulation during growth on GlcNAc for all three time points (Fig. 3A). Quantitative evaluation of the gene expression levels by qPCR in *T. reesei* (Fig. 3B) confirmed a particularly strong induction of the three genes *hxk3*, *dac1* and *dam1*, with 253, 104 and 60‐fold, respectively, after 24 h on GlcNAc compared with the glucose control. In contrast, expression of *nag3*, encoding the GH 3 protein, was only increased 12‐fold and that of the transcription factor *ron1*, 7‐fold. Transcription of the putative GlcNAc‐specific transporter‐encoding gene *ngt1* was increased 40‐fold after 24 h on GlcNAc, indicating involvement in GlcNAc uptake. These data show that the transcription of the genes encoding enzymatic and transport functions in the GlcNAc catabolism pathway is highly induced upon growth on this carbon source, which strongly suggests that the gene cluster is indeed involved in GlcNAc catabolism.

Next, we tested the gene expression of this cluster in *T. atroviride* and *T. reesei* during growth on chitin powder (from crab shells) and colloidal chitin, a better accessible, acid‐pre‐treated form of chitin. Liquid standing cultivations were used, which enable better growth of *Trichoderma* on chitin than shake flask cultivations (Gruber *et al*., 2011). Also under these growth conditions, increased expression of the GlcNAc catabolic gene cluster was detected, although it was less pronounced than on the monomer, GlcNAc (Fig. 3C). Again considerable induction of *hxk3*, *dac1* and *dam1* was observed, while *ron1* and *nag3* were less responsive. In *T. atroviride*, the expression profile of the GlcNAc cluster genes was similar on chitin and colloidal chitin at 48 h and 72 h, while *T. reesei* seemed to respond somewhat slower on untreated chitin and only at 72 h after inoculation, the highest levels of induction of the GlcNAc cluster genes were observed.

### The transcription factor RON1 is essential for the induction of the GlcNAc catabolic gene cluster

Consequently, knockout strains of the GlcNAc catabolic cluster genes in *T. reesei* were generated and functionally analysed. First, single knockout strains were created for *nag3*, *hxk3*, *dac1*, *dam1*, *ron1* and *ngt1* (Table 1) in a Δ*tku70* background, which facilitates homologous integration of gene knockout cassettes due to the lesion in functional non‐homologous end joining (see experimental procedures for details and Fig. S2 for genetic confirmation of the knockout strains). All strains used in this study are listed in Table 1. All subsequent analyses were performed with at least two independent knockout strains. In the figures involving gene deletion strains, the respective results from independent strains and biological replicates are included in the error bars or representative images (e.g. from plates) are presented.

**Table 1:** Fungal strains used in this study

Strain	Genotype	Reference
*T. reesei QM9414*	*mat1‐2*	ATCC 26921
*T. reesei QM9414 Δtku70*	*Δtku70::pyr4 mat 1‐2*	C. Ivanova and B. Seiboth; unpublished data (Ghassemi *et al*., 2015)
*T. reesei Δnag3*	*mat1‐2 Δtku70 Δnag3::hph*	This study
*T. reesei Δhxk3*	*mat1‐2 Δtku70 Δhxk3::hph*	This study
*T. reesei Δdac1*	*mat1‐2 Δtku70 Δdac1::hph*	This study
*T. reesei Δdam1*	*mat1‐2 Δtku70 Δdam1::hph*	This study
*T. reesei Δron1*	*mat1‐2 Δtku70 Δron1::hph*	This study
*T. reesei Δron1 ron1* ^+^	*mat1‐2 Δtku70 Δron1::hph ron1:amdS*	This study
*T. reesei Δngt1*	*mat1‐2 Δtku70 Δngt1::hph*	This study
*T. reesei Δhxk3 G418*	*mat1‐2 Δtku70 Δhxk3::G418*	This study
*T. reesei Δdam1 amdS*	*mat1‐2 Δtku70 Δdam1::amdS*	This study
*T. reesei Δcsp2*	*mat1‐2 Δtku70 Δcsp2::hph*	This study
*T. reesei Δdac1 Δhxk3*	*mat1‐2 Δtku70 Δdac1::hph Δhxk3::G418*	This study
*T. reesei Δdam1 Δhxk3*	*mat1‐2 Δtku70 Δdam1::hph Δhxk3::G418*	This study
*T. reesei Δdac1 Δdam1*	*mat1‐2 Δtku70 Δdac1::hph Δdam1::amdS*	This study
*T. reesei Δhxk3 Δdam1*	*mat1‐2 Δtku70 Δhxk3::hph Δdam1::amdS*	This study
*T. reesei Δhxk3 Δdac1 Δdam1*	*mat1‐2 Δtku70 Δhxk3::G418 Δdac1::hph Δdam1::amdS*	This study

**Figure 4:**
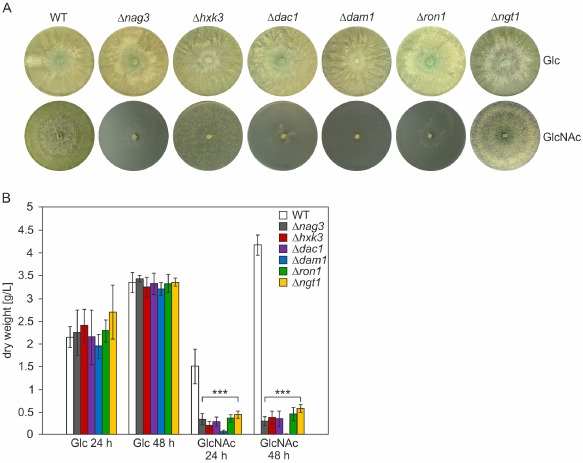
Knockout strains of GlcNAc cluster genes exhibit strongly impaired growth on GlcNAc. A. *T. reesei* strains WT (parental strain; QM9414 Δ*tku70*), Δ*nag3*, Δ*hxk3*, Δ*dac1*, Δ*dam1*, Δ*ron1* and Δ*ngt1* were grown for six days on MA agar plates (1.5% w/v agar) containing either 1% GlcNAc or 1% glucose (Glc) as carbon source. B. For biomass measurements, strains were grown in liquid MA medium in shake flasks, containing either 1% GlcNAc or glucose (Glc) and dry weight was determined after 24 h and 48 h after inoculation. Dry weight of single knockout strains is in all cases statistically significantly different from WT when grown on GlcNAc as carbon source, with *p* < 0.001 for all knockout strains.

All knockout strains exhibited wild‐type growth rate and hyphal morphology as long as GlcNAc (or chitin) was not used as sole carbon source. Carbon sources that were tested are D‐glucose, D‐fructose, cellulose, chitin, glycerol and L‐arabinose. Minimal medium from which a carbon source was omitted was also tested, but did not produce any marked difference in growth compared with the control strain. Figure 4A shows that growth of all deletions strains, except for Δ*ngt1* and Δ*hxk3*, was basically abolished on agar plates with minimal medium [Mandels‐Andreotti (MA)] containing GlcNAc as the carbon source. Δ*hxk3* and Δ*ngt1* strains exhibited sparse outgrowth on agar plates with GlcNAc. Residual growth of Δ*ngt1* strains on agar plates could be due to expression of an alternative transporter that exhibits low affinity for GlcNAc and thereby enables some growth on the added carbon source. In order to assess this possibility, agarose and Phytagel^TM^ (a polymer gelling agent composed of glucuronic acid, rhamnose and glucose units) were also tested as solidifying reagents instead of agar. In each case, residual growth of the Δ*ngt1* strain was observed (data not shown).

Biomass formation of the knockout strains was also quantitatively determined from shake flask cultivations (Fig. 4B). Here, all knockout strains, including Δ*hxk3* and Δ*ngt1*, showed strongly reduced biomass formation on GlcNAc. Therefore, all GlcNAc catabolism cluster genes, including *nag3*, whose function in GlcNAc catabolism remains unclear, appear to be essential for growth on GlcNAc. The putative transporter NGT1 indeed seems to be of major importance for GlcNAc import into the cell. Importantly, Δ*ron1* knockout strains also could not grow on GlcNAc, suggesting that the putative transcription factor RON1 is essential for GlcNAc catabolism. In order to verify our results, complementation strains of *ron1* were generated, which contained an ectopically inserted, functional copy of *ron1* as well as the original deletion of the *ron1* locus (see experimental procedures for details and Fig. S2 for strain verification). Measurements from two independent *ron1*‐complemented strains showed a complete restoration of wild‐type biomass formation (Fig. S3A).

Upon exhaustion of medium nutrients, filamentous fungi undergo autolysis of ageing hyphae. At advanced autolytic growth stages, a decline of biomass can be observed in submerged cultivations. We tested whether a lack of GlcNAc catabolism causes alterations in autolysis due to impaired recycling of chitin from the cell wall. Biomass samples from shake flask cultivations with glucose as carbon source were taken at advanced time points, i.e., 120–216 h after inoculation (Fig. S4). Although onset of autolysis (i.e. the decrease of biomass) appeared delayed in Δ*ron1* compared with the control strain, this difference was not statistically significant. Similar results were obtained for the other knockout strains (data not shown), with the exception of Δ*hxk3* (Fig. S4) which exhibited a statistically significant stronger decrease in biomass than wild type.

**Figure 5:**
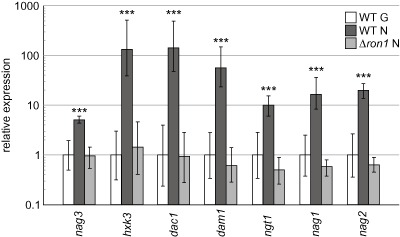
The transcription factor RON1 is the key activator of the GlcNAc catabolic gene cluster. Fungi were grown on MA medium containing 1% glycerol as carbon source for 24 h and then shifted to minimal medium containing 1% GlcNAc (N) or 1% glucose (G) and cultivated for another 24 h. Gene expression levels of all GlcNAc cluster genes, *ngt1* as well as *nag1* and *nag2* were evaluated by qRT‐PCR at 4, 8 and 24 h after the medium shift, normalised using *tef1* as reference gene, and compared with the respective gene expression levels of the parental strain on glucose (control) which was set as 1. The 4, 8 and 24 h values were combined since expression of all three time points was very similar. Statistically significant differences (*P* values < 0.001 were only detected for the parental strain (WT, QM9414 Δ*ku70*) for growth on GlcNAc vs glucose, but not for the Δ*ron1* strain, where gene expression levels on GlcNAc were with relative values around 1 rather similar to those on glucose.

The finding that *ron1* knockout strains could not grow on GlcNAc strongly suggested that RON1 is indeed an indispensable regulator of GlcNAc catabolism. To further confirm that RON1 is a transcriptional regulator of this pathway, transcript levels of the structural GlcNAc catabolic genes were assessed in Δ*ron1* strains. Mycelia were pre‐grown on 1% glycerol and then replaced to either 1% glucose or 1% GlcNAc. Gene expression levels were determined at 4, 8 and 24 h after the replacement and were similar at all three tested time points. The results (Fig. 5) showed that high‐level induction of the genes *hxk3*, *dac1* and *dam1* in the presence of the pathway's substrate, as well as that of *nag3* and *ngt1*, was virtually abolished in *ron1* deletion strains. Gene expression analysis of complemented Δ*ron1* strains (Fig. S3B) showed that an ectopic integration of *ron1* is sufficient to fully restore inducible expression of the other GlcNAc cluster genes in the presence of GlcNAc.

GlcNAc has also been reported to efficiently induce the expression of the GH20 *N*‐acetylglucosaminidase genes *nag1* and *nag2* (López‐Mondéjar *et al*., 2009). Therefore, we analysed whether the lack of *ron1* also influences the expression levels of these genes and found that the induction of *nag1* and *nag2* gene expression by GlcNAc is severely affected in Δ*ron1* strains (Fig. 5). Therefore, these data further substantiated that the transcription factor RON1 is indeed the key activator of the GlcNAc catabolic pathway in *T. reesei*.

### Evaluation of growth conditions related to the GlcNAc catabolism based on observations in other fungi: starvation and regulation by the transcription factor CSP2

Our current study demonstrated for the first time the direct and essential involvement of a fungal Ndt80‐like regulator in the turn‐over of GlcNAc by mediating high‐level induction of the GlcNAc‐catabolic system. For *A. nidulans* XprG mutants, the ortholog of *T. reesei* RON1 (cf. Fig. 2A), a number of observations apparently unrelated to GlcNAc were published. An *xprG* gain‐of‐function mutant (*xprG1*) was described that overproduces extracellular proteases under starvation conditions (Katz *et al*., 2006; 2013). *xprG* loss‐of‐function mutants show reduced extracellular protease levels under both carbon and nitrogen starvation conditions. In addition, HxkC has been described as a non‐catalytic hexokinase‐like protein in *A. nidulans*, likewise modulating the production of extracellular protease (Bernardo *et al*., 2007). However, HxkC is the ortholog of *T. reesei* HXK3; thus, it is likely that HxkC is responsible for the phosphorylation of GlcNAc in *A. nidulans*. We tested growth of knockout strains of the *T. reesei* GlcNAc gene cluster on agar plates containing 1% skimmed milk, the growth condition under which increased protease levels were described for the *xprG* gain‐of‐function mutant. No growth differences (colony diameter or hyphal density) or variation in the diameter of the cleared halo around the growing colonies (indicative of the extracellular protease activity produced) could be detected on MA plates, regardless whether 1% (w/v) skimmed milk powder was used as the sole carbon‐ or as the sole nitrogen source (data not shown). Thus, the lack of *ron1* or *hxk3* or any other gene lesion in GlcNAc catabolism does not appear to affect extracellular protease production in *T. reesei* under these assay conditions.

A survey of the literature for transcription factors that might also be involved in the regulation of GlcNAc metabolism showed that in *N. crassa*, the transcriptional regulator termed GRHL (grainy head like protein) could be involved in the regulation of GlcNAc metabolism in aerial hyphae (Pare *et al*., 2012). On solid medium fungal hyphae grow on and in the substrate and some hyphae can extend into the air (aerial hyphae) and differentiate into specialised structures for the formation of asexual spores (conidia) upon certain stimuli, such as light or nutrient limitation. *N. crassa grhl* mutants are defective in conidial‐spore dispersal in aerial hyphae due to an apparent inability to remodel the cell wall during conidiation. The *grhl* gene is allelic to conidial separation‐2 (*csp‐2*) mutations. The genes of GlcNAc catabolism were among the set of down‐regulated genes in aerial hyphae and conidia from a *N. crassa grhl* mutant (Pare *et al*., 2012).

**Figure 6:**
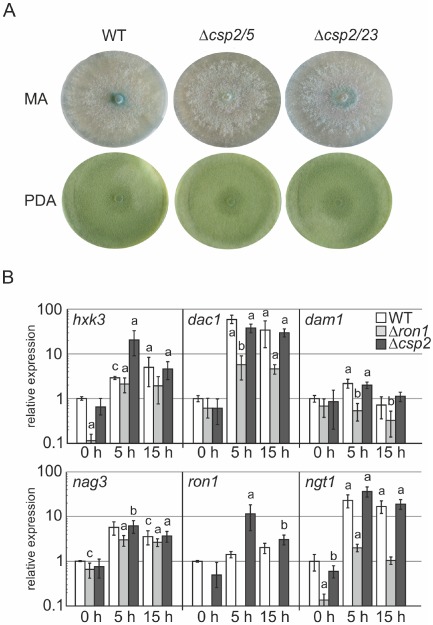
CSP2 is not involved in the regulation of GlcNAc genes during vegetative growth or under carbon starvation conditions. A. Growth of the *T* *. reesei* parental strain QM9414 Δ*tku70* (WT) and two *csp2* knockout strains (Δ*csp2/5* and Δ*csp2/23*) after 7 days on agar plates with MA medium with glucose and PDA. B. qRT‐PCR of the parental strain (WT), and two Δ*ron1* and Δ*csp2* strains. Mycelium was pre‐cultivated for 16 h in MA medium containing 1% glucose, and biomass samples were taken at 0, 5 and 15 h after a shift to MA medium lacking added carbon sources. All measured values were normalised to *tef1* expression and compared with the time point [WT, 0 h], which was set at 1. Bars indicate the SEM (standard error of the mean). ‘a’, ‘b’ and ‘c’ indicate significance at *P* < 0.001, 0.01 and 0.05 respectively.

GRHL proteins belong to the CP2 superfamily of transcription factors (PFAM family PF04516), and in the *T. reesei* genome database, only one predicted member can be found (NCBI accession number: XP_006965082), which we named CSP2. To address its function(s) and assess its relevance for GlcNAc catabolism, we generated knockout strains of *T. reesei csp2* (Fig. S2). *T. reesei* CSP2 and *N. crassa* CSP2 show a high degree of conservation (amino acid similarity: 69%; sequence coverage: 96%). We found that *T. reesei csp2* is expressed under various growth conditions, but we were not able to detect any morphological defects in our Δ*csp2* strains. Analyses included growth on agar plates with either complex medium (potato dextrose agar; PDA) (Fig. 6A) or MA medium with various carbon sources, but colony formation, radial growth rate and sporulation were not altered in Δ*csp2* strains. Further, no phenotype was evident for growth on GlcNAc and chitin (data not shown). Biomass formation in shake flask cultivations was normal for all tested growth substrates. Moreover, microscopical investigation of hyphae or sporulating cultures did not reveal any phenotype for *csp2* knockout strains (data not shown). These data show that, in contrast to *N. crassa*, the sole GRHL‐type transcription factor in *T. reesei* does not seem to have critical functions in conidiation.

To assess potential connections between nutrient limitation and GlcNAc catabolism in more detail, we analysed whether the GlcNAc cluster genes in *T. reesei* are expressed during carbon starvation and whether this expression in the absence of externally supplied GlcNAc might be regulated by RON1 or CSP2. Deletion strains and a wild‐type control strain were pre‐grown for 16 h on 1% glucose and then replaced into medium without carbon source. After the medium shift, samples were taken at different time points (0 h (control), 5 h and 15 h), and expression analysis of the GlcNAc cluster genes was performed. The results showed that in the *T. reesei* parental strain, mainly *dac1* and *ngt1* transcript levels were increased during starvation and that this was dependent on *ron1*, but not on *csp2* (Fig. 6B). In Δ*csp2* strains, some transcript levels were increased transiently at 5 h after the medium shift, e.g. *hxk3* and *ron1*, but it was difficult to discern a trend or a biological consequence for GlcNAc metabolism from that. Interestingly, this experiment also suggested that RON1 antagonises apparent glucose repression of the *ngt1* and *hxk3* genes, i.e. the transporter and the dedicated GlcNAc kinase, directly after the transfer (*t* = 0 h), hence, in the absence of added GlcNAc or a starvation response.

### Conidiation rates in GlcNAc cluster knockout strains

**Figure 7:**
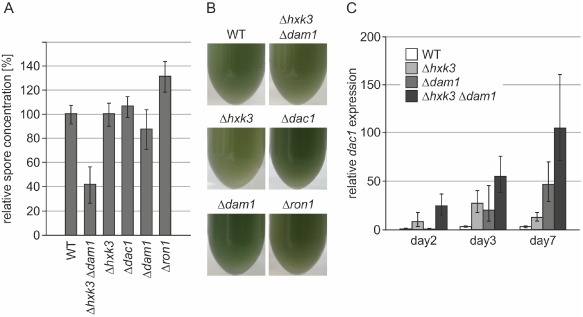
Up‐regulation of *dac1* expression in a Δ*hxk3*Δ*dam1* double knockout strain decreases the amount of produced conidia. A. Percentage of produced conidia in gene knockout strains relative to the parental strain QM9414 Δ*tku70* (WT; 100%). Conidiospore numbers in the Δ*hxk3*Δ*dac1*, Δ*ron1* (*P* < 0.001) and Δ*dam1* (*P* < 0.01) strains are significantly different from the WT. B. Conidiospores were harvested, counted (see Fig. 7A) and diluted to a final spore concentration of 1 × 10^7^ spores ml^−1^. Photographs were taken to visually document the color of the conidia. C. *dac1* expression in spores of different age was assessed in the WT and selected knockout strains (Δ*hxk3*, Δ*dam1* single and Δ*hxk3* Δ*dam1* double knockout). Strains were grown on PDA plates, and spores were harvested after 2, 3 and 7 days after inoculation of the plate for qRT‐PCR analysis, corresponding to nascent, pre‐mature and mature spores of the control strain on PDA plates respectively. Expression levels of *dac1* were compared with WT levels at day 2 and normalised to *tef1* levels. Only the transcription levels of the knock out strains but not the WT levels were significantly different (*P* values < 0.001). This figure is available in colour online at wileyonlinelibrary.com.

We also generated double and triple knockout strains of the GlcNAc cluster genes (Table 1, Fig. S2). Apart from the already observed phenotypes for single knockout strains on GlcNAc, no obvious phenotypic alterations were observed for the double and triple knockout strains except for Δ*hxk3*Δ*dam1* double knockout strains: PDA plates fully overgrown with sporulating colonies of the latter appeared to be ‘less green’, even after incubation for 12 days (data not shown). A more detailed analysis showed that Δ*hxk3*Δ*dam1* strains produced ca. 60% fewer spores/PDA plate than the parental strain (Fig. 7A), but pigmentation of the spores appeared to be normal (Fig. 7B). Spore counting of conidia from 12‐days old PDA plates also revealed that Δ*ron1* produced 30% more conidia than the parental strain and all the other strains of our collection of deletion mutants (Fig. 7A). Microscopic analyses of conidia upon staining of chitin in the fungal cell wall with Calcofluor White did not reveal any differences, and germination rates of the produced conidia were not altered (data not shown). The phenotype of Δ*hxk3*Δ*dam1* strains (which was also verified with another combination of selection marker genes for deletion of *hxk3* and *dam1*, see Table 1, data not shown) was not observed in any of the other combinations of double knockout strains and also not in Δ*hxk3*Δ*dac1*Δ*dam1* triple knockouts. This suggested that the reduced number of conidia could be caused by aberrant *dac1* expression in Δ*hxk3*Δ*dam1* strains and/or a side activity of this enzyme. Gene expression analysis of *dac1* in conidia at different maturation stages showed indeed that this gene is up to 100‐fold up‐regulated in Δ*hxk3*Δ*dam1* double knockout strains compared with the parental strain or the respective single knockout strains (Fig. 7C). These data suggest that the roles of enzymes of the GlcNAc pathway and/or its intermediates could go beyond merely catabolic functions in mobilising reserve nutrients for energy production and nitrogen (re‐)assimilation from cell wall polymers.

## Discussion

The phenomenon of gene clustering has been a powerful aid in the identification, expression and characterisation of fungal secondary metabolism pathways, their intermediates and products (Brakhage, 2013; Wiemann and Keller, 2014; Wisecaver and Rokas, 2015). Clustering of genes appears less frequently in primary metabolism, but both catabolic and anabolic gene clusters have already been described, e.g. for L‐proline catabolism (Jones *et al*., 1981; Hull *et al*., 1989) and D‐biotin biosynthesis (Magliano *et al*., 2011). Comparative genomics revealed that some clusters are quite ancient, while others, such as the ethanol utilisation cluster in certain *Aspergilli* (Flipphi *et al*., 2009), are more recent acquisitions. Clusters can contain structural genes for enzymes, pathway‐related transport functions as well as pathway‐specific regulatory genes, and their exact layout often varies among divergent lineages that share this higher level of organised, functionally related genetic information. In this work, we studied the genomic arrangement of the GlcNAc gene cluster in Ascomycota, generated transcriptional profiles of the respective genes in *T. reesei* and *T. atroviride* and finally characterised them functionally using knockout strains in *T. reesei*. We started by screening the position of the genes encoding homologs of the GlcNAc catabolism pathway in *C. albicans* in sequenced fungal genomes and established the occurrence of a GlcNAc catabolism cluster in all three subphyla of Ascomycota, right down to the most primitive ascomycete (Taphrinomycotina) sequenced to date, *Saitoella complicata* (Fig. 2A). The functional GlcNAc catabolism gene cluster contains two genes in addition to the catabolic enzymes. A gene encoding a GH3 protein (NAG3) is often associated with the four core cluster genes. The *nag3* gene is also present in Saccharomycetales that feature the GlcNAc cluster, as represented by *C. albicans* (not associated with the cluster; Locus CaO19.7516) and *Y. lipolytica* (Fig. 2A). It does not occur in yeasts that miss both the deacetylase and the deaminase, like budding yeast (*S. cerevisiae*) and fission yeast (*S. pombe*). Given the similarity to bacterial exo‐*beta*‐*N*‐acetylhexosaminidases, e.g. *Escherichia coli* NagZ (Litzinger *et al*., 2010), a role in the mobilisation of GlcNAc from various poly and oligomeric precursors appears logical, although we cannot explain why the deletion of *nag3* in *T. reesei* results in a growth phenotype for the monomer (Fig. 4). We are currently studying this gene further to identify its function(s).

The other additional gene that we found in the cluster in comparison to *C. albicans* encodes the transcription factor with an Ndt80‐like DNA‐binding domain that we named RON1. Our *in silico* analysis showed that the GlcNAc cluster in ascomycete filamentous fungi (Pezizomycotina) frequently harbours a gene for this transcription factor. In *C. albicans*, the gene for the Ron1 structural ortholog was previously described as Rep1 and is linked to the GH3 ortholog gene (Fig. 2A). *REP1* is implicated in negative regulation of the multiple drug resistance efflux pump Mdr1: Overexpression in *S. cerevisiae* increased susceptibility to fluconazole, and deletion of *rep1* lead to increased efflux of azole antifungal drugs in *C. albicans* (Chen *et al*., 2009). The documented function of the Ndt80‐like transcription factor Rep1 seems to be different from that of Ron1 in *T. reesei*. We have shown for the first time that an Ndt80‐like transcription factor (RON1) is necessary for growth on GlcNAc as the sole carbon source (Fig. 4) and that it is essential for the high‐level induction of the catabolic genes by GlcNAc (Fig. 5).

GlcNAc catabolism relates directly to nutrient starvation. Polymers of GlcNAc and N‐glycosylated proteins – containing GlcNAc_2_ as a basal anchor of the glycan to the protein – are mobilised to serve as transient carbon and nitrogen sources that allow the fungus to enter asexual reproduction and produce conidia to disseminate and to survive the prevalent unfavourable environmental conditions. Nevertheless, it can be expected that the intracellular GlcNAc pool not only undergoes catabolic recycling but also feeds into anabolism upon phosphorylation to GlcNAc‐6P, followed by isomerisation into GlcNAc‐1P with phospho‐acetylglucosamine mutase (EC 5.4.2.3) and uridylation of the latter into UDP‐GlcNAc with UDP‐GlcNAc pyrophosphorylase (EC 2.7.7.23). The last two enzymes are part of Leloir's UDP‐GlcNAc biosynthetic pathway (for a review in fungi, see Milewski *et al*., 2006). This process provides building blocks for *de novo* chitin synthesis and glycosylation of secreted proteins bypassing the need to re‐synthesise GlcNAc‐6P from fructose‐6P by means of the first two enzymes of UDP‐GlcNAc biosynthesis, GlcN‐6P synthase (EC 2.6.1.16) and GlcN‐6P acetyltransferase (EC 2.3.1.4) (note: these enzymes catalyse the reverse reactions of DAM1 and DAC1 respectively). Indeed, diploid *C. albicans* strains, homozygote for GlcN‐6P synthase deletion could not grow unless the growth medium contained GlcNAc (Gabriel *et al*., 2004).

In filamentous fungi, proteins belonging to the Ndt80‐family have already received considerable attention. RON1 and XprG are orthologs, while VIB‐1‐like proteins are RON1 paralogs unique to Sordariomycetes and Leotiomycetes (Fig. 2B). We therefore speculate that XprG functions and their pleiotropic effects (as evident in *A. nidulans*) could be divided between the RON1‐ and VIB‐1‐like paralogs in species of the two filamentous fungal classes that feature both regulators, such as *T. reesei*, *N. crassa* and *Botrytis cinerea*. The involvement in multiple, seemingly unrelated processes has been reported for VIB‐1, including the regulation of protoperithecial development, vegetative incompatibility and programmed cell death, regulation of responses to nitrogen and carbon starvation, as well as plant cell wall degradation by carbon catabolite repression (Dementhon *et al*., 2006; Hutchison and Glass, 2010; Xiong *et al*., 2014). In the endophytic plant‐symbiont *Epichloë festucae* (Sordariomycetes), VIB‐1 is involved in the production of antifungal compounds (Niones and Takemoto, 2015).

However, none of these studies have revealed the direct connection between Ndt80‐like transcription factors and GlcNAc catabolism or studied GlcNAc as growth substrate although GlcNAc is an important, omnipresent constituent of the fungal cell wall. Additional functions of GlcNAc and its derivatives in signalling and other cellular processes, as reported in *C. albicans*, remain to be elucidated in filamentous fungi.

Our current work strongly suggests that HXK3, the ortholog of *A. nidulans* HxkC (Bernardo *et al*., 2007), is a sugar kinase dedicated to the phosphorylation of GlcNAc, whose induced expression is controlled by RON1, the ortholog of *A. nidulans* XprG (Katz *et al*., 2006). The increased induction levels of the structural GlcNAc catabolic genes in *T. reesei* in response to GlcNAc (Fig. 3) are substantially higher (> 100‐fold) than for any gene mentioned by Katz *et al*. (2013) in their genome‐wide transcriptome analysis of carbon starvation in *A. nidulans*. Nevertheless, one of the best induction ratios comparing xprG^+^ and xprG^−^ strains (6‐fold) was reported for *hxkC*.

Very leaky (starvation) growth of *T. reesei hxk3* knockout strains was observed on GlcNAc agar plates, but not in shake flask cultivations (Fig. 4). This suggests that, although HXK3 is responsible for growth on the acetylated aminosugar, in surface cultures, some phosphorylation can take place in its absence. The enzyme responsible for this activity could be a glycolytic hexokinase; in *T. reesei*, the hexokinase HXK1 and the glucokinase GLK1 involved in the phosphorylation of D‐fructose and D‐glucose, respectively, have been described (Hartl and Seiboth, 2005), but in view of the strong growth defects on many other sugars exhibited by the knockout strains, we refrained from testing this trait further.

An interesting finding concerns simultaneous deletion of *hxk3* and *dam1* that leads to a 60% reduction in condiospore formation in this double knockout mutant and concomitantly, to an up to 100‐fold increase in *dac1* expression in the absence of GlcNAc. In a recent publication, Häkkinen *et al*. found a strong dependence of *dac1* expression on PAC1, the principal mediator of the response to extracellular pH in *T. reesei* (known in *A. nidulans* as PacC: see Peñalva and Arst, 2004). The levels of *dac1* were strongly upregulated in a *pac1* deletion strain, which mimics acidic conditions (Häkkinen *et al*., 2015). A *pac1* deletion in *Trichoderma harzianum* was shown to result in decreased conidiation (Moreno‐Mateos *et al*., 2007). Although we cannot directly explain the correlation between *dac1* up‐regulation and conidiation with our current understanding of GlcNAc catabolism, a pleiotropic effect of elevated *dac1* levels on spore formation cannot be excluded.

In this study, we focussed on functional verification and characterisation of the GlcNAc catabolism pathway and the respective genes in *T. atroviride* and *T. reesei*. Further‐reaching consequences of genetic alterations in this pathway and of its activator RON1 will be the subject of future studies. This could encompass potential feedback effects of the lack of GlcNAc catabolism on chitinase gene regulation as well as more general aspects related to, for example, carbon catabolite repression, as has been reported for *N. crassa* VIB‐1 (Xiong *et al*., 2014). While *Trichoderma* chitinase genes have so far been reported to be inducible only by chito‐oligosaccharides or chitin‐containing polymers (e.g. chitin and fungal cell walls), the two *N*‐acetylglucosaminidase‐encoding genes *nag1* and *nag2* are strongly inducible by the monomer GlcNAc (Mach *et al*., 1999; Gruber and Seidl‐Seiboth, 2012). Although the two proteins NAG1 and NAG2 exhibit different extracellular locations – NAG1 is predominantly secreted into the medium, while NAG2 remains attached to the fungal cell wall; the presence of one of these proteins is both necessary and sufficient to enable growth on chitin (López‐Mondéjar *et al*., 2009). *T. atroviride* cannot grow on the dimer chitobiose when *nag1* and *nag2* genes are absent, which not only shows that there is no additional extracellular *N*‐acetylglucosaminidase that would be able to compensate for NAG1 and NAG2, but also strongly suggests that extracellular cleavage of the dimer into monomers is a prerequisite for uptake in *T. atroviride* (López‐Mondéjar *et al*., 2009). In this study, we could now show that the expression of both *N*‐acetylglucosaminidases is dependent on RON1 since expression of neither of them can be induced in the presence of GlcNAc in a *ron1* deletion mutant. This observation presents an interesting starting point for further investigations of the regulation of chitin metabolism.

As shown in this study, GlcNAc uptake proceeds mainly via the transporter NGT1 because growth of *ngt1* knockout strains was virtually abolished in liquid cultivations. On agar plates, some growth was evident, although the formed mycelium was less dense than in the control strains. The observed weak growth could be explained by presuming the existence of another hexose transporter that has a low affinity for GlcNAc and is expressed sufficiently due to the different type of colony morphology or the different environmental conditions on solid but not in liquid medium. Alternatively, it could be induced by (components of) the solidifying agent to support the observed residual growth on GlcNAc plates.

The GlcNAc gene cluster was not only inducible by GlcNAc, but also by chitin, although induction levels were lower. This is in agreement with the observed slow formation of biomass on chitin and suggests that extracellular depolymerisation is the bottleneck for growth on chitin. For proper understanding of the regulation of chitin degradation from external chitinous carbon (and nitrogen reserve) sources as well as for the process of cell wall remodelling and recycling, knowledge of the GlcNAc catabolic pathway and its possible regulatory and feed‐back functions is pivotal. Chitin degradation narrows down from a multi‐enzyme system at the level of polymeric chitin decomposition to a well‐defined, singular pathway with unique genes for each step. It remains to be elucidated whether altering the flow through the GlcNAc catabolic pathway by manipulation of single genes leads to a better understanding of the regulation and functions of the whole chitin degradation machinery as well as of chitin biosynthesis.

In this study, we progressed towards a more complete insight in fungal chitin degradation by describing the GlcNAc catabolism pathway and its gene cluster in filamentous fungi and established the principle role of the Ndt80 family regulator RON1 as its transcriptional activator.

## Experimental procedures

### Strains and cultivation conditions

*T. atroviride* IMI206040 (ATCC 20476) and *T. reesei* QM9414 (ATCC 26921) were used in this study and maintained on potato dextrose agar (PDA, BD, Franklin Lakes, USA). Agar plates were kept in the dark at 28°C for *T. reesei* or in a 12 h light/12 h dark cycle at 28°C for *T. atroviride*. Stock cultures were kept in 50% glycerol at −80°C. For yeast transformation to generate deletion constructs, the shuttle vector pRS426 (Christianson *et al*., 1992) and the yeast strain WW‐YH10 (ATCC 208405) were used. *E. coli* Stellar™ Competent Cells (Clontech Laboratories, Mountain View, CA) were used for propagation of all used and constructed plasmids.

For shake flask cultivations MA medium [pH 5; per liter: 1.4 g (NH_4_)_2_SO_4_, 2.0 g KH_2_PO_4_, 0.3 g MgSO_4_*7H_2_O, 0.3 g CaCl_2_*2H_2_O, 5 mg FeSO_4_*7H_2_O, 1.6 mg MnSO_4_*H_2_O, 1.4 mg ZnSO_4_*7H_2_O and 2 mg CoCl_2_*2H_2_O for *T. reesei* (adapted from (Mandels and Andreotti, 1978)] and SM medium for *T. atroviride* (Seidl *et al*., 2004) with 0.05% peptone and 1% glucose (D‐Glucose Monohydrate; Roth, Karlsruhe, Germany) or *N*‐acetylglucosamine (GlcNAc; Sigma‐Aldrich, St. Louis, MO) as carbon source was inoculated with 1 × 10^6^ conidia ml^−1^ and cultivated at 28°C and 220 r.p.m. for the indicated time periods. For replacement of carbon sources, *T. reesei* strains were pre‐grown in MA medium containing 0.05% peptone and 1% glycerol (for replacement with GlcNAc and glucose) or 1% glucose (for starvation) for 24 h and 16 h, respectively, harvested by filtration through sterile filter tissue (Miracloth; Calbiochem, Merck, Darmstadt, Germany), washed with sterile tap water and transferred to fresh MA medium, containing carbon sources as stated in the figure legends in the results section and again incubated at 28°C, 220 r.p.m. for the indicated time periods. Static liquid cultivations were performed in sterile Petri dishes (100 mm diameter, Greiner Bio‐one, Kremsmünster, Austria) with MA medium containing 1% glucose, powdered chitin (Sigma‐Aldrich) or colloidal chitin, inoculated with 1 × 10^6^ conidia ml^−1^ and cultivated at 28°C. Colloidal chitin was prepared as described in Seidl *et al*. (2005). Mycelial samples from shake flask and static cultures were harvested by filtration through Miracloth (Calbiochem, Merck), washed with cold distilled water and immediately frozen in liquid nitrogen. Mycelial dry weight was determined by withdrawing 50 ml aliquots from shake flask cultivations, suction filtration through a glass wool filter, followed by extensive washing with tap water, and drying at 80°C to constant weight.

For preparation of conidia from different maturation stages, based on the appearance of the mycelium covered with conidia, ranging from white (2 days) to light green (3 days) and dark green conidia (7 days) strains were grown on PDA plates in the dark at 28°C. Conidia were harvested at the indicated time points by rinsing the sporulating mycelium with a 0.9% NaCl/0.05% Tween‐80 solution, which was then transferred into a sterile 2 ml reaction tube, concentrated by centrifugation (2,500 *g*, 3 min), and the conidial pellet was immediately frozen in liquid nitrogen.

For spore counting strains were grown for 12 days on PDA. Conidiospores were harvested from plates in a defined volume of 0.9% NaCl/0.05% Tween‐80 solution/plate and counted in a Thoma‐cell counting chamber (Roth). The amount of total spores per ml was calculated using the formula given by the manufacturer. To visually determine the color of the mature spores, a dilution to 1 × 10^7^ spores per ml was produced according to the spore concentration determined by cell counting. Statistical analysis for growth tests and spore generation was performed using the Student's *t*‐test, assuming unequal variance of groups.

### Isolation and purification of fungal DNA and RNA

For RNA isolation, the samples were ground with mortar and pestle to a fine powder under liquid nitrogen, and total RNA was isolated using the guanidinium thiocyanate method (Sambrook and Russell, 2001). Isolated RNAs were treated with DNAse I (Fermentas, St Leon‐Roth, Germany), and cDNAs were subsequently generated with the Revert Aid H‐minus cDNA synthesis kit (Fermentas). For verification of knockouts, genomic DNA was isolated using a rapid DNA purification protocol (Liu *et al*., 2000). Genomic DNA from *T. reesei* was isolated using the Wizard Genomic DNA Purification Kit (Promega).

### Gene expression analysis (RT‐PCR and quantitative RT‐PCR)

RT–PCR (17‐30 cycles depending on the probe, see Table S1) was performed using the gene‐specific primers listed in Table S1 along with the database accession numbers of the genes analysed in this study. The *tef1* gene (JGI protein ID 83874 for *T. reesei* and JGI protein ID 300828 for *T. atroviride* in the JGI database; NCBI database: XP_006964056 and EHK42777 respectively) was used as the reference gene. qRT‐PCR reactions were performed in a Bio‐Rad (Hercules, CA, USA) iCycler IQ. The reaction mix contained 12.5 μl SYBR green Supermix (Bio‐Rad), 8.5 μl pure water, 6.25 μM forward and 6.25 μM reverse primer, and 2 μl 1:50 diluted template cDNA. For cDNA synthesis, 5 μg of RNA/reaction were reverse‐transcribed using the Revert Aid H‐minus cDNA synthesis kit (Fermentas). Primer efficiency for qPCR was calculated using a dilution series from 1:5 to 1:5000 with the PCR baseline‐subtracted mode. Amplification efficiency was then calculated from the given slopes in the IQ5 Optical system Software v2.0. The amplification protocol consisted of an initial denaturation step for 3 min at 95°C, followed by 40 cycles of denaturation (95°C for 15 s), annealing and elongation (60°C for 15 s). Oligonucleotides are listed in Table S1. The *tef1* gene was used as a reference. Relative gene expression ratios were calculated using REST software (Pfaffl *et al*., 2002). For analyses of the results, samples from at least two independent experiments with three technical replicates in each run were used. The significance of differences in gene expression between different knockout mutants and the parental strain as well as between cultivation conditions at a given time point was evaluated by the Student's *t*‐test, assuming unequal variance of groups.

### Generation of *T*. *reesei* gene knockout strains

Gene deletions were performed in a *T. reesei* QM9414 Δ*tku70* strain in which the *tku70* gene was replaced by the *pyr4* marker (Ghassemi *et al*., 2015; C. Ivanova and B. Seiboth, unpub. results). Effects of the deletion of *tku70* in *T. reesei* have been described in Guangtao *et al*. (2009). Gene knockout cassettes were generated using the yeast recombination system described in Schuster *et al*. (2012). For generation of the 5′‐ and 3′‐flanking sequences consisting of around 1 kb of up‐ and downstream non‐coding regions of the respective genes, primers were designed according to Schuster *et al*. (2012). Sequences were obtained from the *T. reesei* genome database (http://genome.jgi‐psf.org/Trire2/Trire2.home.html). Primers for 5′‐ and 3′‐flanking sequences for the knockout cassette construction are listed in Table S2. As selectable marker the hygromycin phosphotransferase gene *hph*B from *E. coli* was used to generate single knockout strains (Mach *et al*., 1994). For generation of double and triple knockout strains, the geneticin resistance gene (*npt2*, from *E. coli* (Bischof and Seiboth, 2014)) and acetamidase gene (*amd*S, from *Aspergillus nidulans*; described for application as selection marker in *Trichoderma* in Penttilä *et al*., 1987) were used as selectable markers (the primers for amplification of the marker genes are listed in Table S3). In the course of these experiments, also single knockout strains with *npt2* (*hxk3*) and *amdS* (*dam1*) markers were generated (Table 1) with which also the results from the *hph*B marker deletion strains could be verified. The amplified 5′ and 3′ flanking regions of the respective GlcNAc cluster genes and the marker genes were inserted in pRS426 by homolog recombination in yeast (Schuster *et al*., 2012). Transformation of *T. reesei* was performed essentially as described in Gruber *et al*. (1990). Correct integration of the gene deletion cassette was verified by PCR (primers are listed in Table S4) after two rounds of purification by single spore isolation on selective medium containing 0.1% Triton X‐100. Selective media were PDA with 100 μg ml^−1^ Hygromycin B (Roth) or 120 μg ml^−1^ Geneticin (G418‐Sulfat, Roth) for *hphB* and *npt2*, respectively, and 0.1 M acetamide (Merck) in MA medium lacking other nitrogen sources for *amdS*. A replacement strain of the Δ*ron1* strain was constructed by heterologously reintroducing the wild‐type *ron1* gene coupled to an *amdS* cassette into the constructed Δ*ron1* strains. Therefore, *ron1* was amplified from genomic *T. reesei* DNA, assembled with the *amdS* cassette downstream of the gene in the pRS426 vector and transformed into the Δ*ron1* mutant strains as described above. Table 1 shows all generated knockout strains and the Δ*ron1 ron1* ^+^ replacement strain. Knockout strains and the Δ*ron1 ron1* ^+^ replacement strain were verified for correct integration of the cassette by PCR (Fig. S2) and by absence of the wild‐type band of the respective target genes and gene expression (data not shown). At least two independent knockout strains for each of the analysed genes were used for characterisation.

### Microscopic analyses

For detailed morphological characterisation of knockout strains, liquid cultures supplemented with the carbon sources indicated in the respective results sections were incubated for 24, 48 and 72 h; 200 μl of each culture was placed on a microscope chambered coverglass (Thermo Fisher Scientific) and imaged with an inverted Nikon T300 microscope (Nikon, Tokyo, Japan) with differential interference contrast optics and a DXM1200F digital camera (Nikon). For microscopical investigation of the chitin content of fungal cell walls, they were incubated with a solution containing Calcofluor White to a final concentration of 2.5 μM and imaged on a Nikon C1 confocal laser scanning unit containing a 405 nm laser mounted on a Nikon Eclipse TE2000 inverted microscope.

### Bioinformatic analyses

For analysis of the GlcNAc catabolic gene cluster in filamentous fungi, genomic data of the 18 token species of Ascomycota depicted in Fig. 2A, accessible at the National Centre for Biotechnology Information (NCBI), were mined for orthologs of the six genes involved in GlcNAc catabolism that are described in this paper, i.e. *hxk3*, *dac1*, *dam1*, *ngt1*, *ron1* and *nag3*, by TBLASTN screening (Altschul *et al*., 1990). The query proteins used were the functional *C. albicans* structural proteins Nag1 (*T. reesei* DAM1), Nag2 (*T. reesei* DAC1), Nag5 (*T. reesei* HXK3) and Ngt1 (*T. reesei* NGT1) (all of these, encoded by intron‐less genes), the predicted GH family 3 protein from *T. reesei* (NAG3, protein ID: 79669, NCBI accession number: XP_006966911) and *A. nidulans* (NCBI TPA accession number CBF84813; 923 aa) and the Ndt80‐like regulator PhoG/XprG from *A. nidulans* (NCBI TPA accession number CBF84810: 588 aa), as well as its ortholog in *T. reesei* RON1 (protein ID: 79673, NCBI accession number: XP_006966820). Gene models were determined manually, and clustering and orientation were subsequently deduced for the closely linked genes. Intergenic spaces within (sub)clusters were checked for (additional) reading frames.

To study the evolutionary relations among ascomycete proteins harbouring an *NDT80*‐like DNA‐binding domain, we mined the NCBI Whole Genome Shotgun contigs database – including an ample range of species of Eurotiomycetes, Sordariomycetes and Dothideomycetes – for the encoding genes in Pezizomycotina and Taphrinomycotina and the NCBI Reference Genomic Sequences database for the encoding genes in Saccharomycetales. As queries for TBLASTN screening, we used the characterised regulatory proteins *Saccharomyces cerevisiae* Ndt80 (Xu *et al*., 1995), *Neurospora crassa* Vib1 (Xiang and Glass, 2002), *Aspergillus nidulans* PhoG/XprG (version TPA Accession CBF84810; (Wortman *et al*., 2009) and its *T. reesei* ortholog, RON1 (Kubicek *et al*., 2011). Gene models and products were deduced manually, in some cases with the aid of publicly available EST sequences, for instance, for the correct 586 residues‐long Ndt80‐ortholog in *Neurospora crassa*, GenBank accessions GE976053 and BG280050. PhoG/XprG‐ and Vib1‐like proteins were assigned to two groups (tagged _1 and _2 respectively) based on the gene model (intron‐exon structure) of the encoding genes, while Ndt80‐like proteins (labelled _3) are clearly distinct. In the subphylum Taphrinomycotina, we only found a PhoG/XprG‐like protein specified in the primitive species *Saitoella complicata*. Two paralog Ndt80‐like proteins deduced from the genomes of seven strains of *Rhizophagus irregularis*, belonging to the phylum of Glomeromycota, were included to provide an evolutionary anchor that eventually appeared at the basis of the branch of the Ndt80‐like (group 3) ascomycete proteins.

Approximately 500 resulting protein sequences from this search were aligned with MAFFT version 7 (Katoh and Toh, 2008) using the E‐INS‐i algorithm and a BLOSUM 45 similarity matrix. The alignment was curated with Block Mapping and Gathering using Entropy (Criscuolo and Gribaldo, 2010) using a BLOSUM 40 similarity matrix and a block size of 3, yielding 135 informative residues per protein. A maximum likelihood tree was then calculated with the PhyML program applying the WAG substitution model (Guindon *et al*., 2010) and drawn with FigTree (available at http://tree.bio.ed.ac.uk/software/figtree). The drawing software allows simplification of branches or clades to group phylogenetically related proteins with retention of species information in cartoons (Fig. S1) as well as collapsing branches or clades to the level of entire classes or entire families (Fig. 2B) (NB: all known Saccharomycotina belong to the order of Saccharomycetales). Hence, Fig. 2B shows an extremely compressed version while Fig. S1 is a more extensive, albeit still simplified, reflection of the same maximum likelihood tree. Approximate likelihood ratio tests (Anisimova and Gascuel, 2006) were calculated integrally by PhyML using Chi2‐based parametric, and aLRT values (0–1) are given at the connecting nodes in the tree.

## Supporting information

Supporting information
