# Supplementary material for: The N‐acetylglucosamine catabolic gene cluster in Trichoderma reesei is controlled by the Ndt80‐like transcription factor RON1
Source: Mol Microbiol. 2015 Nov 19;99(4):640–57. doi: 10.1111/mmi.13256 (PMC4950302; doi:10.1111/mmi.13256)
Supplement: Supplementary file 1 — Supporting information [file MMI-99-640-s001.pdf]

Supplementary Information

The *N*-acetylglucosamine catabolic gene cluster in *Trichoderma reesei* is controlled by the Ndt80-like transcription factor RON1

Lisa Kappel[1], Romana Gaderer[1], Michel Flipphi[2] and Verena Seidl-Seiboth[1]

[1] Research Division Biotechnology and Microbiology, Institute of Chemical Engineering, TU Wien, Vienna, Austria

[2] Department of Biochemical Engineering, Faculty of Science and Technology, University of Debrecen, Debrecen, Hungary

Corresponding author:

Verena Seidl-Seiboth, Research Division Biotechnology and Microbiology, Institute of Chemical Engineering, TU Wien, Gumpendorfer Strasse 1a, 1060 Vienna, Austria. Tel.: +43-1-58801-166554, Fax: +43-1-58801-17299. Email: verena.seidl@tuwien.ac.at

Running Title: *N*-acetylglucosamine catabolism in *Trichoderma*

Key words: chitin, *N*-acetylglucosamine, transcription factor, gene cluster, *Trichoderma*

This PDF file includes:

Supplementary Tables S1 to S4

Supplementary Figures S1 to S4

Supplementary Tables

Table S1: RT-PCR and qRT-PCR primers

T. atroviride RT-PCR primers

Gene	JGI - ID	Oligo	sequence 5´-3´	number of cycles
nag3	138324	Ta_GH3_RTs	CCTCGAAGGCTCACCAACAT	25
		Ta_GH3_RTa	TCAAATGCCTGCAGAACATCG	
hxk3	223428	Ta_HEX_RTs	CAGAACCTCAACGTCGAGCT	25
		Ta_HEX_RTa	CGTGGGATGAAGGATGCTTCT	
dac1	79361	Ta_DAC_RTs	GGCATCATCTACTCGGTGGG	28
		Ta_DAC_RTa	GTGGGATTCCGGTGCCTG	
dam1	257866	Ta_DAM_RTs	ACATCCACCCTTCCAACGTC	25
		Ta_DAM_RTa	ATCTGCTCGAATCCAGCCTC	
ron1	284947	Ta_TF_RTs	GCGAAGAAACACAAGCAGCA	25
		Ta_TF_RTa	GGGCGATCGTGTGATGAACT	
tef1	300828	Ta_tef1_RTs	GGTACTGGTGAGTTCGAGGCTG	17
		Ta_tef1_RTa	GGGCTCAATGGCGTCAATG	

T. reesei RT-PCR primers

Gene	JGI - ID	Oligo	sequence 5´-3´	number of cycles
nag3	79669	Tr_GH3_RTs	ATCTGAGTGCCTGGTATGCG	25
		Tr_GH3_RTa	CCACGAGCGGCTCATACG	
hxk3	79677	Tr_HEX_RTs	GGGCAAGATCTGGGAGACAT	25
		Tr_HEX_RTa	TGGGATGAAGGGTACTTTTCCG	
dac1	79671	Tr_DAC_RTs	CATCACCTACTCGGTCGGC	28
		Tr_DAC_RTa	CCTGAGGGCGTGAGGAATG	
dam1	79674	Tr_DAM_RTs	GCCTACGAGGACGCCATTAA	25
		Tr_DAM_RTa	CCTTGGACGGCAGGATCTG	
ron1	79673	Tr_TF_RTs	GCGGGACAGTCTACTTGCTT	25
		Tr_TF_RTa	TGAGCCTTGAATTCTGGGGG	
ngt1	80863	Tr_NGTL_RTs	ACAACCTGCTGTACTGGCTC	25
		Tr_NGTL_RTa	CGGCGAACATGGTGTCGTA	
csp2	60761	Tr_CSP2_RTs	GGTCGAGTGGAATAAGCAAGG	30
		Tr_CSP2_RTa	GATGGCCGATGTTTGGGAC	
tef1	46958	Tr_tef1_Fw	GTACTGGTGAGTTCGAGGCTG	17
		Tr_tef1_Rev	GGGCTCGATGGAGTCGATG	

T. reesei qRT-PCR primers

Gene	JGI - ID	Oligo	sequence 5´-3´
nag3	79669	Tr_GH3_1qPCRs	GGTTGTGGACGATGCGTAT
		Tr_GH3_1qPCRa	CGTTTCGGGGGAGTTTGTT
hxk3	79677	Tr_HEX_2qPCRs	CCGAGGTGGGACAATACTT
		Tr_HEX_2qPCRa	GATGCGGTAGTTTTGTATGCT
dac1	79671	Tr_DAC_2qPCRs	CTTCAACTCGCATCCCGAC
		Tr_DAC_2qPCRa	GGAGCCCACCTTGACAAT
dam1	79674	Tr_DAM_2qPCRs	CGTAAACATCCACCCGTCCAA
		Tr_DAM_2qPCRa	CTCTTAATGGCGTCCTCGT

ron1	79673	Tr_TF_2qPCRs	GCCCGTCACCAATCAAGA
		Tr_TF_2qPCRa	CTCCCGCCTCATCCACAATA
ngt1	80863	Tr_NGTL_1qPCRs	TCTTGTTCGTCCTCACCTTT
		Tr_NGTL_1qPCRa	CATCCTCCCAGTCAACCTT
tef1	46958	tef1_qPCR_fw	CCACATTGCCTGCAAGTTCGC
		tef1_qPCR_rv	GTCGGTGAAAGCCTCAACGCAC

Table S2 - Oligos for deletion of GlcNAc catabolic cluster genes, *ngt1* and *csp2* as well as oligos for heterologous *ron1* reintroduction

Gene	JGI - ID	Oligo	sequence 5´-3´
nag3	79669	5F_TR_GH3	GTAACGCCAGGGTTTTCCCAGTCACGACGACAGTAGTAGAATGCCGTGC
		5R_TR_GH3_hph	CATATTGATGTAAGGTAGCTCTCGGATCCCCGTCTACAGTATCACCTCC
		3F_TR_GH3_hph	TATTCCATCTAAGCCATAGTACCCTCGAGCACCGAAAAGTACACTCACG
		3R_TR_GH3	GCGGATAACAATTTCACACAGGAAACAGCCGAGGCATCATCTACTATGG
hxk3	79677	5F_TR_HEX	GTAACGCCAGGGTTTTCCCAGTCACGACGTCCTCCCTTTACTTCTCTCG
		5R_TR_HEX_hph	CATATTGATGTAAGGTAGCTCTCGGATCCGACTCTAGGACATGAGCAGG
		3F_TR_HEX_hph	TATTCCATCTAAGCCATAGTACCCTCGAGTCACAGTTGATGCTCTCAGC
		3R_TR_HEX	GCGGATAACAATTTCACACAGGAAACAGCGACCTCGGAGAAGATCATGG
		5R_TR_HEX_g418	CTGGGAACAACTGGCATGAATTCATCGATGACTCTAGGACATGAGCAGG
		3F_TR_HEX_g418	TAGAGGTAATCCTTCTTTCTAGAGGATCCTCACAGTTGATGCTCTCAGC
dac1	79671	5F_TR_DAC	GTAACGCCAGGGTTTTCCCAGTCACGACGAGTTCAGTTCCATCCGTAGC
		5R_TR_DAC_hph	CATATTGATGTAAGGTAGCTCTCGGATCCTCTTACACACCACTCACACC
		3F_TR_DAC_hph	TATTCCATCTAAGCCATAGTACCCTCGAGATAGTCGCAGTCTGTTCTGC
		3R_TR_DAC	GCGGATAACAATTTCACACAGGAAACAGCGGAGTCCCTACCTAGTATCG
dam1	79674	5F_TR_DAM	GTAACGCCAGGGTTTTCCCAGTCACGACGACATGCAGTACAGTACCACC
		5R_TR_DAM_hph	CATATTGATGTAAGGTAGCTCTCGGATCCCTACTACATGCACCCTATCC
		3F_TR_DAM_hph	TATTCCATCTAAGCCATAGTACCCTCGAGGCACGTCATACTTGTACTCG
		3R_TR_DAM	GCGGATAACAATTTCACACAGGAAACAGCTCAGAGGACGAAAAGAGTCC
		5R_TR_DAM_amdS	GATGACATTCATACTCAAGACGTCGACCTCTACTACATGCACCCTATCC
		3F_TR_DAM_amdS	CGCTTCCATCTCTCAAAGGAAGAATCCCTGCACGTCATACTTGTACTCG
ron1	79673	5F_TR_TF	GTAACGCCAGGGTTTTCCCAGTCACGACGACCTAGTAGATACCGAGTGC
		5R_TR_TF_hph	CATATTGATGTAAGGTAGCTCTCGGATCCTGAGAGACGAGATGACAACG
		3F_TR_TF_hph	TATTCCATCTAAGCCATAGTACCCTCGAGCGAGTACAAGTATGACGTGC
		3R_TR_TF	GCGGATAACAATTTCACACAGGAAACAGCCCATGACATTAGCAAGGTGC
	ron1$^+$	5R_RPM_TR_TFamdS	GATGACATTCATACTCAAGACGTCGACCTCCATGACATTAGCAAGGTGC
	(RPM)	amds_RPM_TF_F	CCATGCGAGGCACCTTGCTAATGTCATGGAGGTCGACGTCTTGAGTATG
		amdS_RPM_TF_R	GCGGATAACAATTTCACACAGGAAACAGCAGGGATTCTTCCTTTGAGAG
ngt1	80863	5F_TR_NGTL	GTAACGCCAGGGTTTTCCCAGTCACGACGTACCTCTGTCTCGTATCTGG
		5R_TR_NGTL_hph	CATATTGATGTAAGGTAGCTCTCGGATCCCGGACAGTGAAGAAGAAAGC
		3F_TR_NGTL_hph	TATTCCATCTAAGCCATAGTACCCTCGAGCAGCTGTTTGAACTGAGACC
		3R_TR_NGTL	GCGGATAACAATTTCACACAGGAAACAGCTCGGTACAAGCTGTAAGACC
csp2	60761	5F_TR_CSP2	CGGTACCCGGGGATCCCCTGAGTCTCTCTGGTCAGCC
		5R_TR_CSP2_hph	GGTAGCTCTCGGATCCGTCTCTCGACTCTTTTCACC
		3F_TR_CSP2_hph	GCAGGCATGCAAGCTTCTTCTACCCCTTCTTCTTCC
		3R_TR_CSP2	TGATTACGCCAAGCTTGCTTCAGACTACAACTGTCC

RPM… primers used for heterologous replacement with *ron1*

Table S3 - Primers and vectors used for amplification of gene knockout cassettes

Primer	sequence 5´- 3´	amplicon size [bp's]
hph F selbst	GGATCCGAGAGCTACCTTAC	2359
hph R selbst	CTCGAGGGTACTATGGCTTA	
amds F	AGGTCGACGTCTTGAGTATG	3841
amds R	AGGGATTCTTCCTTTGAGAG	
npt2/G418 F	ATCGATGAATTCATGCCAGTTGT	2837
npt2/G418 R	GGATCCTCTAGAAAGAAGGATTACCT	

Table S4 - Primers for verification of knockout strains

Primer	sequence 5´- 3´
GH3_ko_ctrl_F	AAAAAGCCAGCAGAAACGGC
HEX_ko_ctrl_F	CCTTGAGGGCATGTCTTGGT
DAC_ko_ctrl_F	GCAGCACGGCTAGGTACCTA
DAM_ko_ctrl_F	TGCTGTTAGGTACTGTGGCC
TF_ko_ctrl_F	GGAGGAGGAAGAGGAGGAGG
TF_RPM_ ctrl_F	GCGGGACAGTCTACTTGCTT
NGTL_ko_ctrl_F	CCGCCTGAGATATGGAGTGG
CSP_ko_ctrl_F	GTGGGCATGTACGGATCTCAG
Hph_ko_ctrl_R	TTGCGAGGCTGGTGATTAGG
G418_ko_ctrl_R	TCCGGGTTTTCGAGTCGTTT
AmdS_ko_ctrl_R	GCCGAGTTAGCCGAAATTGG

Supplementary Figures

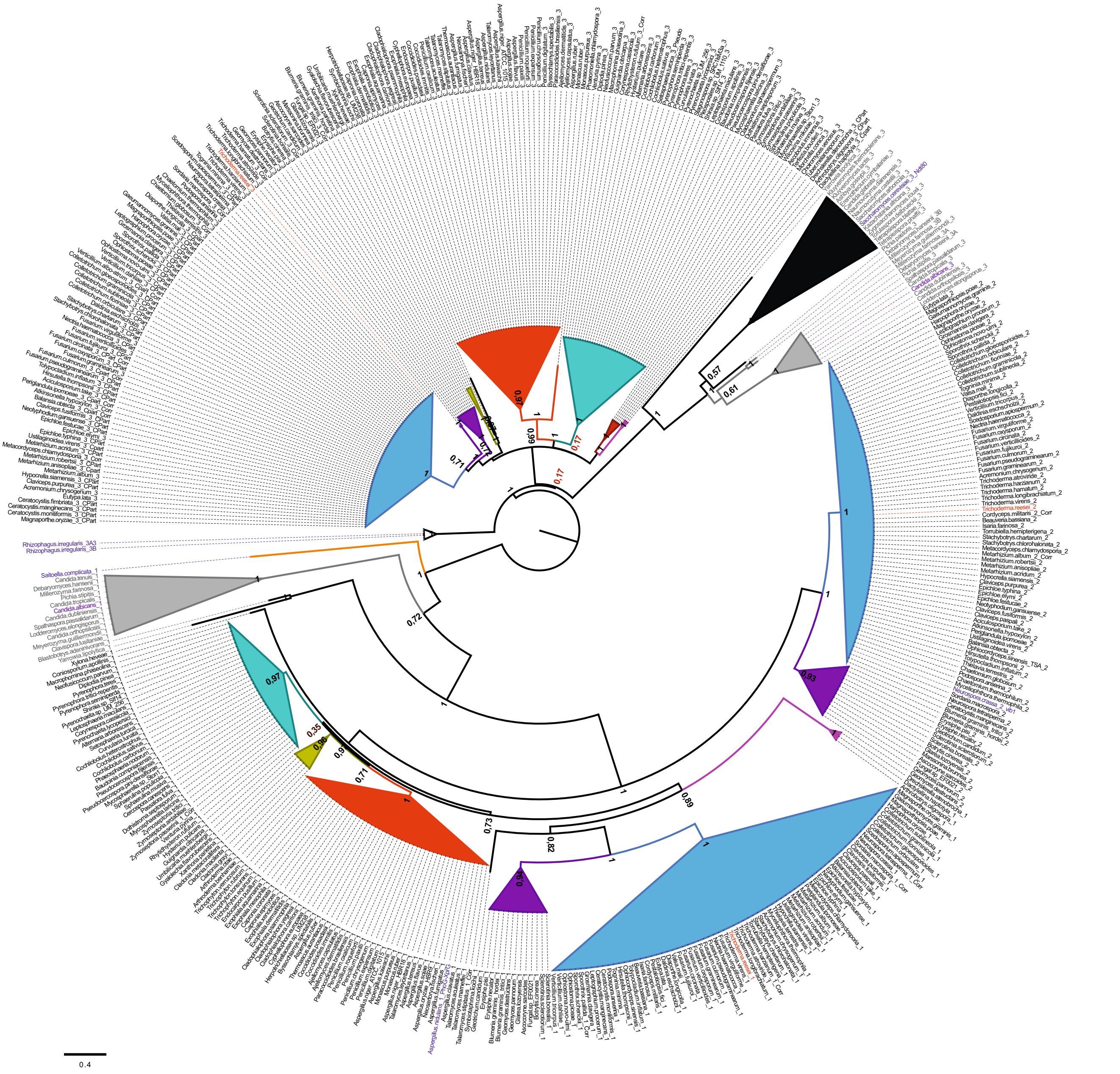

Fig. S1: Phylogenetic analysis of proteins with an Ndt80-like domain in Ascomycota (expanded tree). This figure shows an extended maximum likelihood tree in circular format representing the same analysis on which the compressed tree shown in Fig. 2B is based. To facilitate readability, clades of related proteins were grouped in "cartoons" rather than collapsed to entire classes (for Pezizomycotina) or families (for Saccharomycetales): Sordariomyces, light blue; Leotiomycetes, purple; Eurotiomycetes, red; Dothideomycetes, turquiose; Lecanoromycetes, olive green; Orbiliomycetes, pink; Pezizomycetes (only group 3 proteins), auburn; Saccharomycetaceae (only group 3 proteins), black; Debaryomycetaceae, grey. The branch of the *Saitoella complicata* group 1 protein, unique among Taphrinomycotina, is drawn orange and appears at the basis of the RON1/XprG/VIB1 clade (protein_1 and protein_2, ca. 290 proteins). The two Ndt80-like proteins in *Rhizophagus irregularis* (phylum Glomeromycota) anchor the Ndt80 clade (protein_3, ca. 210 proteins). The three proteins from *T. reesei* are highlighted in red letters. The two proteins from *C. albicans* are highlighted in purple letters. The names of (the other) Saccharomycotina species are printed in grey. The three characterized proteins *S. cerevisiae* Ndt80, *A. nidulans* PhoG/XprG and *N. crassa* VIB-1, are highlighted in blue letters, as are the proteins from *S. complicata* and *R. irregularis* at the basis of the two superbranches.

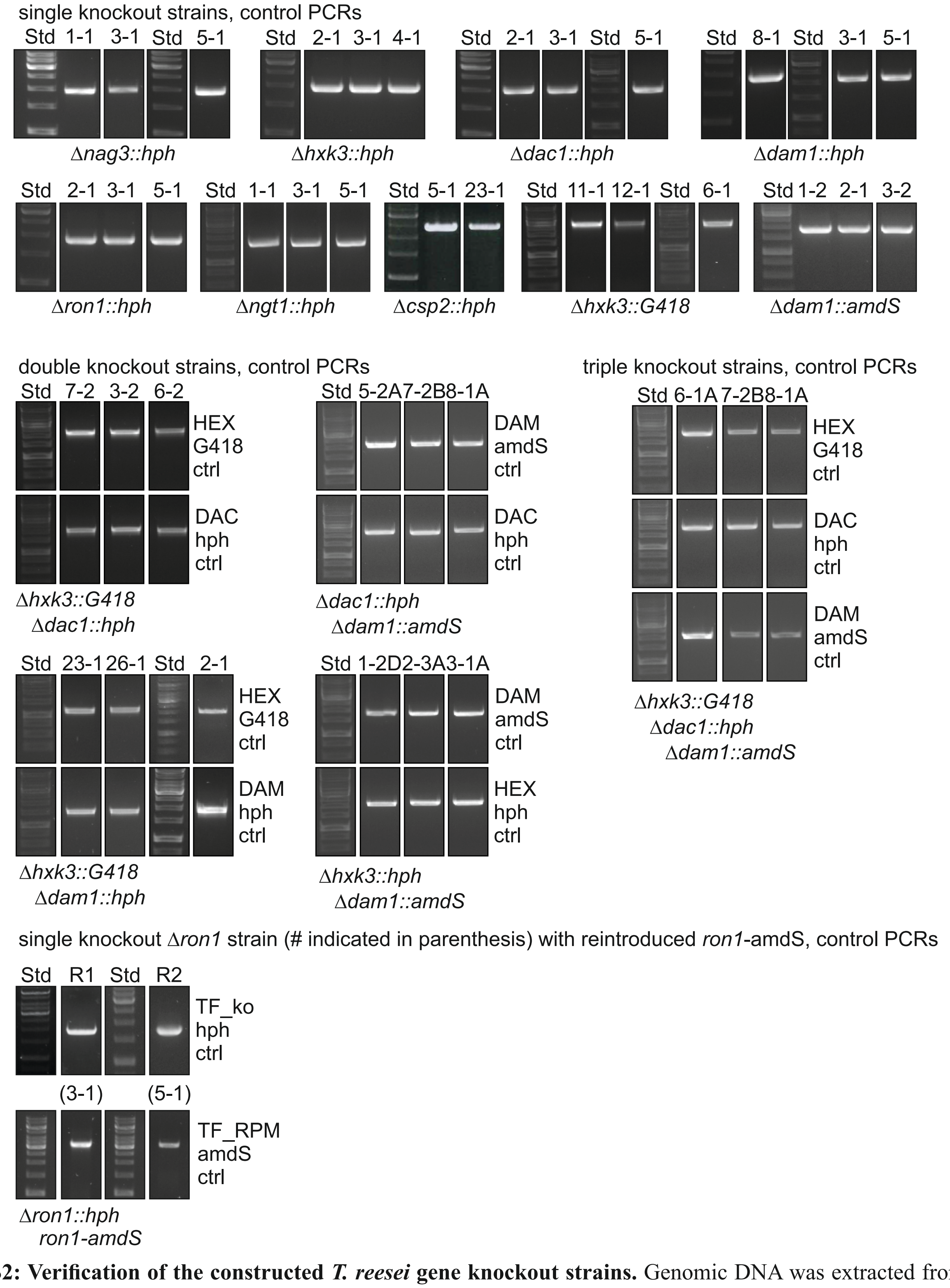

Fig. S2: Verification of the constructed *T. reesei* gene knockout strains. Genomic DNA was extracted from purified single, double and triple knockout strains as described in the experimental procedures section and subjected to PCR with primers binding to a sequence in the selection marker cassette and a sequence upstream of the 5′ flanking region of the deleted gene, respectively (Table S4), generating an approximately 2 kb long PCR product in knockout strains. For the construction of the Δ*ron1 ron1*$^{+}$ replacement strain Δ*ron1* (3-1) and (5-1) strains were chosen for heterologously reintroducing the *ron1::amdS* construct. In double and triple knockout strains as well as the Δ*ron1 ron1*$^{+}$ replacement strain, which were generated from extant single deletion strains, all expected gene deletions were again verified. (Std) Gene Rule, 1 kb DNA Ladder (Fermentas).

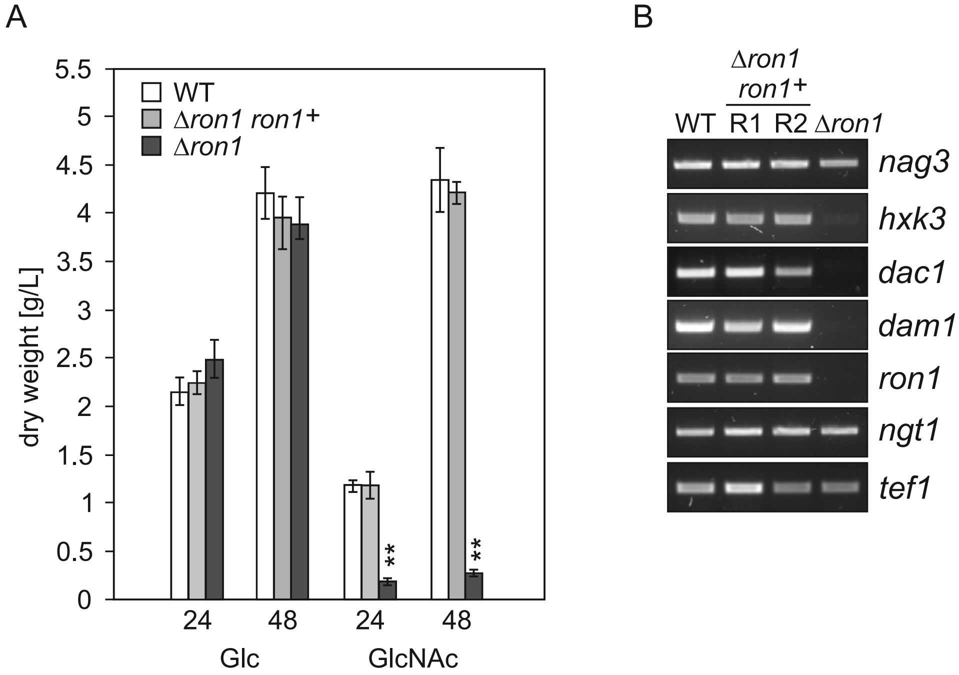

Fig. S3: Reintroduction of *ron1* into a *ron1* deleted strain rescues the defects observed for growth on GlcNAc. (A) For biomass measurements, the parental strain (WT, QM9414 Δ*tku70*), Δ*ron1* and Δ*ron1 ron1*$^{+}$ (R1 and R2), were grown in liquid MA medium in shake flasks, containing either 1% GlcNAc or glucose (Glc) and dry weight was determined after 24 h and 48 h of growth. Only the dry weight of Δ*ron1* strains is statistically significantly different from WT when grown on GlcNAc as carbon source, with $p < 0.01$. (B) Gene expression analysis (semi-quantitative RT-PCR) of the parental strain (WT), Δ*ron1* and Δ*ron1 ron1*$^{+}$ (R1 and R2) after pre-growth on glycerol (1%) for 24 h and a shift to 1 % GlcNAc medium for 4 h. As reference gene *tef1* was used. Note that substantial basal levels of *nag3* and *ngt1* transcript are present in the Δ*ron1* mutant strain.

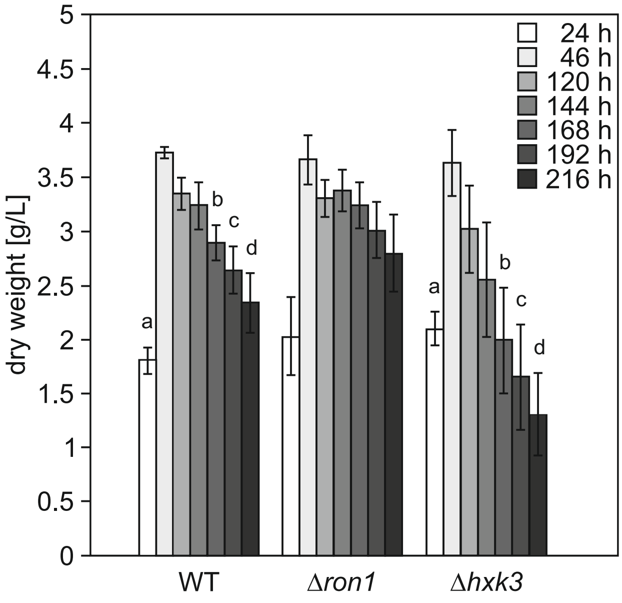

Fig. S4: Deletion of the *hxk3* gene significantly influences autolysis upon carbon starvation. Biomass measurements of *T. reesei* strains QM9414 Δ*tku70* (WT), Δ*ron1* and Δ*hxk3* grown in liquid minimal medium in shaking flasks, containing 1% glucose (at the time of inoculation). Mycelial dry weight was determined after 24, 46, 120, 144, 168, 192 and 216 h of growth. The means of 2 independent experiments and two different transformants are shown. Dry weight differs only significantly between WT and Δ*hxk3* mutant at 24 (a), 168 (b), 192 (c) and 216 h (d) (a-c: $p < 0.05$; d: $p < 0.01$).
